# p53 codon 72 polymorphism in vulval cancer and vulval intraepithelial neoplasia

**DOI:** 10.1054/bjoc.2000.1419

**Published:** 2000-10-26

**Authors:** A N Rosenthal, A Ryan, D Hopster, I J Jacobs

**Affiliations:** Department of Gynaecological Oncology,Academic Department of Histopathology, St. Bartholomew's and the Royal London School of Medicine and Dentistry, Queen Mary and Westfield College, West Smithfield, London, EC1A 7BE, UK

**Keywords:** VIN, vulval cancer, p53, polymorphism

## Abstract

p53 codon 72 polymorphism was analysed in UK women with human papillomavirus (HPV)-associated vulval intraepithelial neoplasia and vulval squamous cell carcinoma. Arginine homozygotes were significantly less common in either group compared with controls. We conclude that the arginine polymorphism may confer protection against the development of HPV-associated vulval neoplasia. © 2000 Cancer Research Campaign


British Journal of Cancer (2000) **83**(10), 1287–1290
© 2000 Cancer Research Campaign
doi: 10.1054/ bjoc.2000.1419, available online at http://www.idealibrary.com on IDEAL®

Short Communication

### p53 codon 72 polymorphism in vulval cancer and vulval intraepithelial neoplasia

**AN Rosenthal[1], A Ryan[1], D Hopster[2] and IJ Jacobs[1]**

[1]Department of Gynaecological Oncology; [2]Academic Department of Histopathology, St. Bartholomew's and the Royal London School of Medicine and Dentistry, Queen Mary and Westfield College, West Smithfield, London EC1A 7BE, UK

**Summary** p53 codon 72 polymorphism was analysed in UK women with human papillomavirus (HPV)-associated vulval intraepithelial neoplasia and vulval squamous cell carcinoma. Arginine homozygotes were significantly less common in either group compared with controls. We conclude that the arginine polymorphism may confer protection against the development of HPV-associated vulval neoplasia. © 2000 Cancer Research Campaign

**Keywords:** VIN; vulval cancer; p53; polymorphism

Recent in vitro functional data (Storey et al, 1998) suggests that the arginine variant at codon 72 of the p53 tumour suppressor gene is more susceptible to degradation by oncogenic human papillomavirus (HPV) E6 protein than the proline variant. Preliminary epidemiological data (Storey et al, 1998) found that women with cervical cancer were more likely to be homozygous for arginine than healthy controls. However, several larger studies from Europe (Hayes et al, 1998; Helland et al, 1998; Josefsson et al, 1998; Lanham et al, 1998; Rosenthal et al, 1998), the USA (Hildesheim et al, 1998; Sonada et al, 1999), Japan (Wang et al, 1999) and China (Minaguchi et al, 1998; Ngan et al, 1999*a*) have failed to reproduce this finding. Nevertheless, one large European study (Zehbe et al, 1999) has found an excess of arginine homozygotes in cervical cancer patients from both Italy and Sweden compared with healthy controls. A weaker association was found with pre-invasive disease.

Studies of this polymorphism in the context of HPV infection have to date been limited to cervical cancer. Recent studies in head and neck cancer (Hamel et al, 2000) and skin cancer (Marshall et al, 2000) found no association between the polymorphism and malignancy, however neither study tested for HPV status, even though HPV is implicated in only a proportion of these cancers. As vulval cancer and intraepithelial neoplasia are often associated with HPV infection, we decided to investigate this polymorphism in UK women with vulval intraepithelial neoplasia (VIN) and invasive vulval squamous cell carcinoma (VSCC). While the vast majority of VIN is associated with oncogenic HPV types (Kohlberger et al, 1998), this is not the case with VSCC, in which only 15–57% of samples may contain HPV DNA (Lee et al, 1994; Kagie et al, 1997). We therefore initially tested our formalin-fixed tissue samples for the presence of genital HPV types.

*Received 9 November 1999*
*Revised 14 June 2000*
*Accepted 26 June 2000*

*Correspondence to:* IJ Jacobs

### MATERIALS AND METHODS

### Populations studied and extraction of DNA

Samples containing both normal and neoplastic tissue (either VIN, VSCC or both) from UK caucasian women were retrieved from the pathology archives of St. Bartholomew's and the Royal London hospitals. The relevant paraffin-embedded tissue samples were serially sectioned as follows: one 4-micron section was mounted, stained with haematoxylin and eosin (H+E), covered and used as a reference slide. One 10-micron section was also stained with H+E, but left uncovered. The uncovered 10-micron section was mounted on a dissecting microscope and compared with the reference slide. Areas of tissue containing > 70% VSCC, VIN or normal tissue were identified by a histopathologist and microdissected. DNA extraction was performed in 100 µl of 10% Chelex chelating resin (Sigma, Saint Louis, MS, USA) and 200 µg $ml^{-1}$ proteinase K (Boehringer Mannheim, Lewes, UK), vortexed, placed in a shaking water bath at 56°C for 30 min and boiled for 8 min to inactivate the enzyme. The sample was centrifuged at 10 000 ***g*** for 10 min to pellet any remaining debris. 1–4 µl of supernatant was used directly in the polymerase chain reaction (PCR). The control population used was that described in our previous study (Rosenthal et al, 1998). Briefly, this comprised DNA extracted from 96 caucasian women from different parts of the UK taking part in a randomized controlled trial of ovarian cancer screening and 150 caucasian women attending for routine antenatal care in the Oxford region of the UK. The p53 codon 72 genotype frequencies did not differ significantly between these two groups and so they were combined for the purpose of statistical comparison with the study groups, as in our original study (Rosenthal et al, 1998).

### PCR amplification of consensus genital-type HPV L1 gene

PCR in a Touchdown Thermal Cycler (Hybaid, Asford, UK) was performed in 20 µl containing 0.2 mM deoxyribonucleotide triphosphates, 0.25 U of Taq supreme DNA polymerase (Hellena

Biosciences, Sunderland, UK), 1 × buffer (supplied with enzyme) and 20 pmol each of primers GP5+ and GP6+ (Kohlberger et al, 1998). PCR was performed using a 3 min denaturation step at 94°C followed by 40 cycles of denaturation at 94°C for 1 min, annealing at 48°C for 2 min and extension at 72°C for 1.5 min. A final extension step at 72°C for 7 min was performed. Hela cell DNA was used as positive control and cross-contamination was checked for using water controls. PCR products were electrophoresed on a 2% agarose gel and visualized using ethydium bromide staining and ultraviolet illumination.

### PCR amplification of p53 codon 72 polymorphic alleles

PCR in a Touchdown Thermal Cycler (Hybaid, Asford, UK) was performed in a volume of 20 μl containing approximately 20–100 ng DNA extracted from microdissected normal tissue, 80 μM deoxynucleotide triphosphates, 0.1 U of Red Hot DNA polymerase (Advanced Biotechnologies, Epsom, UK), 1 × buffer (supplied with enzyme), 1.5 mM $Mg^{2+}$, 1.5 pmol of forward primer, 2 pmol of reverse primer and 0.5 pmol of $^{32}$P-labelled forward primer. Primers for the arginine allele have been previously described (Storey et al, 1998). Primers for the proline allele were as follows: 5′-GCCAGAGGCTGCTCCCCC and 5′-GAAGGGACAGAAGATGACAGG. PCR for the arginine allele was performed using a 5 min denaturation step at 94°C followed by 35 cycles of denaturation at 94°C for 1 min, annealing at 54°C for 1 min and extension at 72°C for 1 min. A final extension step at 72°C for 5 min was performed. PCR for the proline allele was performed using the same conditions as those for the arginine allele, except for a different annealing temperature of 58°C. Homozygous arginine and proline controls were used. The PCR products were electrophoresed on a 5% denaturing polyacrylamide gel. Autoradiography was performed. The arginine-specific PCR results in a 141 base-pair product and the proline-specific PCR results in a 101 base-pair product. The reliability of this assay was confirmed by direct sequencing of products amplified from the control samples from our original study (Rosenthal et al, 1998).

### Statistical analysis

95% confidence intervals for the observed genotype frequencies were calculated. The chi-squared test was used to establish whether the sample and control groups were in Hardy–Weinberg equilibrium and whether the proportions of the three p53 codon 72 genotypes differed between the sample and control groups.

### RESULTS

We identified 48 cases of VIN not associated with VSCC and 52 cases of VSCC which were HPV-positive. The results of the analysis are shown in Figure 1, along with the results of the control populations from our previous study (Rosenthal et al, 1998). There were significant differences in the proportions of the three p53 codon 72 genotypes (arginine homozygotes, proline homozygotes and heterozygotes) for both vulval cancer vs controls (df = 2, $P = 0.0023$, $\chi^2 = 12.11$) and for VIN vs controls (df = 2, $P < 0.0001$, $\chi^2 = 22.21$). These differences resulted from a lower frequency of arginine homozygotes in both the vulval cancer group (42%) and the VIN group (29%) compared with the control group (63%). The control population was in Hardy–Weinberg

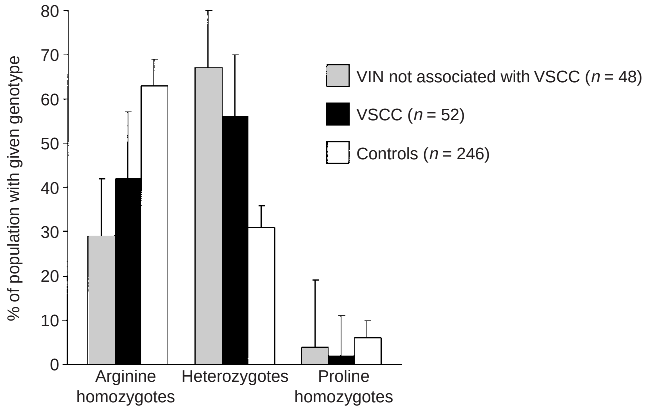

**Figure 1** Distribution of p53 codon 72 alleles in women with VIN, women with VSCC and healthy controls (error bars = 95% confidence intervals)

equilibrium (df = 1, $P = 0.123$, $\chi^2 = 2.38$), but neither the vulval cancer nor the VIN populations were in equilibrium (vulval cancer df = 1, $P = 0.016$, $\chi^2 = 5.76$; VIN df = 1, $P = 0.003$, $\chi^2 = 8.56$).

### DISCUSSION

If women homozygous for the arginine variant of the p53 codon 72 polymorphism are at increased risk of cervical cancer (Storey et al, 1998), then this has important implications for targeting cervical screening to a high-risk population. As the polymorphism was thought to increase cervical cancer risk by rendering p53 product more susceptible to HPV E6-mediated degradation, the arginine variant might also be expected to increase the risk of other anogenital HPV-associated neoplasias. Included in this group are VIN and some VSCC, in which the predominant HPV is the oncogenic type 16 (Hording et al, 1993; Lee et al, 1994; Kagie et al, 1997; Ngan et al, 1999*b*). We therefore tested the hypothesis that HPV-positive VIN and VSCC were associated with being homozygous for the arginine variant. If such an association were found, such women might benefit from increased surveillance. This study is the first to examine the relationship between p53 codon 72 polymorphism and proven HPV-associated neoplasia outside the cervix.

We demonstrated a significant association between the p53 codon 72 polymorphism genotype and the type of sample studied. However, in contrast to the two previous positive studies in cervical cancer (Storey et al, 1998; Zehbe et al, 1999), we observed a lower frequency of arginine homozygotes in the HPV-associated vulval cancer and VIN patients than in healthy controls. This suggests that arginine homozygotes have a lower risk of developing vulval neoplasia, compared with women who have one or more proline alleles. This is supported by the observation that neither the VSCC nor the VIN population were in Hardy–Weinberg equilibrium, indicating that the presence of a proline allele appears to select in favour of the vulval neoplasia phenotype. We are unaware of any biologically plausible reason as to why the arginine variant might confer protection, but it is interesting to note that studies of lung (Birgander et al, 1995) and nasopharyngeal cancer (Birgander et al, 1996) have also demonstrated an association between the proline allele and disease. However, the risk of the proline allele applied mostly in combination with the presence of a 16 base-pair duplication in intron 3 of the p53 gene. This suggests that the proline allele may be a marker

 © 2000 Cancer Research Campaign for linkage disequilibrium with a functional cancer susceptibility site elsewhere in the gene.

Eleven of 13 different populations reported in the 11 studies so far published have failed to demonstrate an association between p53 codon 72 polymorphism and cervical cancer, while two have found a positive association between this disease and the arginine variant. There are three possible explanations for the conflicting results in these studies. First, accidental inclusion of HPV-negative cancers in some of the studies could have diluted the true study population. This criticism is not relevant in cervical cancer where almost all squamous cell carcinomas (SCC) are associated with HPV (Walboomers et al, 1999). It could be argued that of the oncogenic HPV types, only HPV 16 and 18 E6 proteins have been shown to degrade the arginine variant of p53 more easily than the proline variant (Storey et al, 1998) and that other types of oncogenic HPV may not exhibit this property. However approximately 75% of cervical SCC in Europe contain these two types of HPV (Bosch et al, 1995), so this is an unlikely explanation. In the case of vulval cancer, not all cases are HPV-positive, so we used sensitive consensus primers to detect genital HPV types and only performed the polymorphism analysis in HPV-positive cancers. Previous studies (Hording et al, 1993; Lee et al, 1994; Kagie et al, 1997; Ngan et al, 1999*b*) have demonstrated that the vast majority of HPV-positive VSCC contains type 16.

A second possible explanation for discrepant results in polymorphism studies is accidental inclusion of people from different ethnic groups. We were careful to ensure that only UK caucasian women were included in both our previous study and this study. The other studies of this polymorphism describe ethnic matching of cases and controls. It is interesting to note that the UK caucasian control population from the original pilot study (Storey et al, 1998) had different allele frequencies to the larger UK caucasian populations in subsequent studies (Rosenthal et al, 1998; Lanham et al, 1998). While the pilot study control population allele frequencies may not have been statistically in disequilibrium, this may reflect the relatively small sample size.

The third possible explanation for discrepant results is the accuracy of the polymorphism assay used. While we cannot comment on different methods used by other groups, our previous report (Rosenthal et al, 1998) used the same technique as that used in the pilot study (Storey et al, 1998). We modified this technique in the current study because the frequency of loss of heterozygosity at the p53 locus in VSCC or VIN has not yet been reported. It is therefore possible that performing the polymorphism assay on samples containing neoplastic tissue could underestimate the proportion of true heterozygotes if some of them had lost one copy of the p53 locus. We therefore tested microdissected normal tissue. It was necessary to redesign one of the primers for amplification of the proline allele in order to yield a relatively smaller product, which could be reliably amplified from the lower concentrations of DNA obtained from microdissected tissue. Direct sequencing of this product confirmed that it amplified the proline variant.

In conclusion, the balance of evidence suggests that the arginine polymorphism, while functionally different from the proline in vitro, does not appear to be associated with an increased risk of cervical neoplasia. Even if much larger studies were to refute this conclusion, the high frequency of arginine homozygotes in Northern European populations means that this polymorphism is unlikely to be useful as a test to identify women at higher risk of cervical cancer in such populations. In contrast to the evidence in cervical cancer, our data suggests that the arginine variant may protect against the development of HPV-associated vulval neoplasia.

### ACKNOWLEDGEMENTS

We would like to thank Mr Alex Brown and his staff for cutting and mounting the tissue slides, Mr Steve Jones for providing the list of patients with VIN and VSCC and Dr Steven Skates for assisting with the statistical analysis. ANR was supported by the Gynaecology Cancer Research Fund and AR was supported by the Stroyberg Vagn Jensen foundation.

### REFERENCES

Birgander R, Sjalander A, Rannug A, Alexandrie AK, Sundberg MI, Seidegard J, Tornling G, Beckman G and Beckman L (1995) P53 polymorphisms and haplotypes in lung cancer. *Carcinogenesis* **16**: 2233–2236

Birgander R, Sjalander A, Zhou Z, Fan C, Beckman L and Beckman G (1996) p53 polymorphisms and haplotypes in nasopharyngeal cancer. *Hum Hered* **46**: 49–54

Bosch FX, Manos MM, Munoz N, Sherman M, Jansen AM, Peto J, Schiffman MH, Moreno V, Kurman R and Shah KV (1995) Prevalence of human papillomavirus in cervical cancer: a worldwide perspective. International biological study on cervical cancer (IBSCC) study group. *J Natl Cancer Inst* **87**: 796–802

Hamel N, Black MJ, Ghadirian P and Foulkes WD (2000) No association between p53 codon 72 polymorphism and risk of squamous cell carcinoma of the head and neck. *Br J Cancer* **82**: 757–759

Hayes VM, Hofstra RMW, Buys CHCM, Hollema H and van de Zee AGJ (1998) Homozygous arginine-72 in wild type p53 and risk of cervical cancer. *Lancet* **352**: 1756

Helland A, Langerod A, Johnsen H, Olsen A, Skovlund E and Borresen-Dale A-L (1998) p53 polymorphism and risk of cervical cancer. *Nature* **396**: 830–831

Hildesheim A, Schiffman M, Brinton LA, Fraumeni JF Jr, Herrero R, Bratti MC, Schwartz P, Mortel R, Barnes W, Greenberg M, McGowan L, Scott DR, Martin M, Herrera JE and Carrington M (1998) p53 polymorphism and risk of cervical cancer. *Nature* **396**: 531–532

Hording U, Kringshome B, Andreasson B, Visfeldt J, Daugaard S and Bock JE (1993) Human papillomavirus in vulvar squamous-cell carcinoma and in normal vulvar tissue: a search for a possible impact of HPV on vulvar cancer prognosis. *Int J Cancer* **55**: 394–396

Joseffson AM, Magnusson PKE, Ylitalo N, Quarforth-Tubin P, Ponten J, Adami HO and Gyllensten UB (1998) p53 polymorphism and risk of cervical cancer. *Nature* **396**: 531

Kagie MJ, Kenter GG, Zomerdijk-Nooijen Y, Hermans J, Schuuring E, Timmers PJ, Trimbos JB, and Fleuren GJ (1997) Human papillomavirus infection in squamous cell carcinoma of the vulva, in various synchronous epithelial changes and in normal vulvar skin. *Gynecol Oncol* **67**: 178–183

Kohlberger P, Kainz C, Breitenecker G, Gitsch G, Sliutz G, Kolbl H, Tschachler E and Reinthaller A (1998) Absence of p53 protein overexpression in precancerous lesions of the vulva. *Cancer* **82**: 322–327

Lanham S, Campbell I, Watt P and Gornall R (1998) p53 polymorphism and risk of cervical cancer. *Lancet* **352**: 1631

Lee YY, Wilnczynski SP, Chumakov A, Chih D and Koeffler HP (1994) Carcinoma of the vulva: HPV and p53 mutations. *Oncogene* **9**: 1655–1659

Marshall SE, Bordea C, Wojnarowska F, Morris PJ and Welsh KI (2000) p53 codon 72 polymorphism and susceptibility to skin cancer after renal transplantation. *Transplantation* **69**: 994–996

Minaguchi T, Kanamori Y, Matsushima M, Yoshikawa H, Takateni Y and Nakamura Y (1998) No evidence of correlation between polymorphism at codon 72 of p53 and risk of cervical cancer in Japanese patients with human papillomavirus 16/18 infection. *Cancer Research* **58**: 4585–4586

Ngan HY, Liu VW and Liu SS (1999*a*) Risk of cervical cancer is not increased in Chinese carrying homozygous arginine at codon 72 of p53. *Br J Cancer* **80**: 1828–1829

© 2000 Cancer Research Campaign 

Ngan HY, Cheung AN, Liu SS, Yip PS and Tsao SW (1999*b*) Abnormal expression or mutation of TP53 and HPV in vulvar cancer. *Eur J Cancer* **35**: 481–484
Rosenthal AN, Ryan A, Al-Jehani RM and Jacobs IJ (1998) p53 codon 72 polymorphism and risk of cervical cancer in UK. *Lancet* **352**: 871–872
Sonada Y, Saigo PE and Boyd J (1999) p53 and genetic susceptibility to cervical cancer. *J Natl Cancer Inst* **91**: 557
Storey A, Thomas M, Kalita A, Harwood C, Gardiol D, Mantovani F, Breuer J, Leigh IM, Matlewski G and Banks L (1998) Role of a p53 polymorphism in the development of human papillomavirus-associated cancer. *Nature* **393**: 229–234
Walboomers JMM, Jacobs MV, Manos MM, Bosch FX, Kummer JA, Shah KV, Snidjers PJ, Peto J, Meijer CJ and Munoz N (1999) Human papillomavirus is a necessary cause of invasive cervical cancer worldwide. *J Pathol* **189**: 12–19
Wang NM, Tsai CH, Yah KT, Chen SJ and Chang JG (1999) p53 codon 72Arg polymorphism is not a risk factor for cervical cancer in the Chinese. *Int J Mol Med* **4**: 249–252
Zehbe I, Voglino G, Wilander E, Genta F and Tommasino M (1999) Codon 72 polymorphism of p53 and its association with cervical cancer. *Lancet* **354**: 218–219

 *© 2000 Cancer Research Campaign*

